# Ablation Behavior of a Carbon Fabric Reinforced Phenolic Composite Modified by Surface-Decorated ZrB_2_/SiC

**DOI:** 10.3390/ma13020256

**Published:** 2020-01-07

**Authors:** Feng Xu, Shizhen Zhu, Jingdan Hu, Zhuang Ma, Yanbo Liu

**Affiliations:** 1 School of Material Science and Engineering, Beijing Institute of Technology, Beijing 100081, China; sdxfeng2013@163.com (F.X.); florahjd@163.com (J.H.); hstrong929@bit.edu.cn (Z.M.); boobbyy@163.com (Y.L.); 2 National Key Laboratory of Science and Technology on Material under Shock and Impact, Beijing 100081, China

**Keywords:** carbon fabric reinforced phenolic composite, surface-decorated ZrB_2_–SiC, mechanical performance, ablation behavior

## Abstract

Carbon fabric reinforced phenolic composites were widely used as TPSs (thermal protection system) material in the aerospace industry. However, their limited oxidative ablation resistance restricted their further utility in more serious service conditions. In this study, the surface-decorated ZrB_2_/SiC and its modified carbon fabric reinforced phenolic composites have been successfully prepared. The self-modification mechanism of the surface-decorated ZrB_2_/SiC particles were characterized. The mechanical performance and ablation behavior of the composites were investigated. Results showed that the ZrB_2_/SiC particles possessed a good surface-decorated effect, which achieved good compatibility with the phenolic resin. The mechanical performance of the modified phenolic composite was effectively improved. The anti-oxidative ablation performance of the composite was improved. The mass ablation rate of the surface-decorated ZrB_2_–SiC-modified carbon fabric reinforced phenolic composites was 25% lower than that of the unmodified composites. The formed ZrO_2_ ceramic layer attached to the surface of the residual chars prevented the heat energy and oxygen from the inner material. Meanwhile, the volatilization of SiO_2_ and B_2_O_3_ effectively increased the heat dissipation. All these results confirmed that the ZrB_2_–SiC particles can effectively improve the ablation resistance of the composite, which provided a basis for the application of the composites to more serious service environments.

## 1. Introduction

The serious heating flow formed by the intense friction between a hypersonic vehicle and the surrounding air severely affects the flight stability of an aircraft [1,2,3,4]. Carbon fabric reinforced phenolic (C–Ph) composites possessing high thermal stability and good mechanical property is an ideal ablative heat-preventing material, which was widely used for thermal protection systems [5,6,7,8]. However, with the development of the aerospace industry, the ablation resistance of the C–Ph composite made it difficult to meet the requirements for more serious service conditions. Therefore, using appropriate materials to effectively improve the anti-ablation performance of the C–Ph composite is of great significance. 

Nowadays, many researches focused on the organic–inorganic hybrid modification using single non-oxide ceramic particles [9,10,11,12]. Different kinds of these particles have been used for enhancing the ablation resistance of composites. Ding et al. [10] introduced zirconium silicide into carbon-phenolic composites. The ZrSi_2_ was oxidized to form the ZrO_2_ and SiO_2_ during the ablation process, which effectively enhanced the ablation resistance of the C–Ph composites. George et al. [11] investigated the thermal stability of SiC-modified phenolic resin composites. They found that SiC had a positive effect on the matrix phenolic resin, which enhanced the mechanical property and thermo-stability of the composites. Chen et al. [12] studied the ablation mechanism of ZrB_2_-modified C–Ph composites. They discovered that the existence of ZrB_2_ remarkably improved the ablation resistance of the C–Ph composites due to the formation of ZrO_2_. Although, all the hybrids exhibited positive characteristics of the added non-oxide ceramic components. It is still difficult to achieve a continuous wide-temperature-range protection during the ablation process. 

The multiphase ultra-high temperature ceramic ZrB_2_–SiC was widely used in the field of anti-oxidative ablation for ceramic matrix composites [13,14,15,16,17]. Both ZrB_2_ and SiC have special properties in high temperature, such as a great thermal shock resistance, high melting temperature and great chemical inertness. Yang et al. [13] studied the anti-ablation mechanism of the ZrB_2_–SiC coating for Cf/SiC composites. They found that the composite possessed better anti-ablative properties. During the ablation process, ZrB_2_–SiC was oxidized to form B_2_O_3_, ZrO_2_ and SiO_2_.The evaporation of B_2_O_3_ and SiO_2_ can take away a large amount of heat and the melted SiO_2_ can effectively seal the holes to obstruct the oxygen [14,15,16]. The formed B_2_O_3_, SiO_2_ and ZrO_2_ can realize continuous wide-temperature-range protection during the ablation process. Therefore, all these indicate that ZrB_2_–SiC is an ideal material to be used for anti-oxidative ablation for resin matrix composites.

In this paper, the ZrB_2_–SiC particles were chemically grafted with 3-aminopropyltriethoxysilane (APS) to have a surface decoration. The purpose of the surface decoration is to achieve the organic–inorganic hybridization of the ZrB_2_–SiC particles and further improve the compatibility between the resin and the particles. Then, different content of surface-decorated ZrB_2_–SiC particles was added to modify the phenolic resin and prepared the composites by compression molding. The self-modification mechanism of the surface-decorated ZrB_2_/SiC particles were investigated. The anti-ablation behavior and mechanism of the composite were analyzed.

## 2. Materials and Methods

### 2.1. Materials

Phenolic resin (solid content of 80%, SW-802) was supplied by the Institute of Chemistry, Chinese Academy of Sciences. Carbon fabric (CF, PAN-based epoxy resin-sized T300, thickness of 0.1 mm) was purchased from Xiangsheng CF Technology (Yancheng, Jiangsu) Co., Ltd., China. ZrB_2_ and SiC (1–3 μm, 98%, Forsman Scientific (Beijing) Co., Ltd., China) were used as inorganic additives. 3-aminopropyltriethoxysilane (APS, 99 wt %) was purchased from Macklin (Beijing) Co., Ltd., China.

### 2.2. Preparation of Surface-Decorated ZrB_2_–SiC-Modified Phenolic Composites 

Firstly, the ZrB_2_–SiC particles were mixed by mechanical stirring with a volume ratio of 7:3. Then these particles were mixed with absolute ethanol, APS and deionized water according to the ratio as listed in Table 1. The mixture was heat refluxed at 80 °C for 10 h, 15 h and 20 h, respectively. Subsequently, the treated particles were washed respectively and dried in an oven at 80 °C to obtain the surface-decorated ZrB_2_–SiC. Next, the surface-decorated ZrB_2_–SiC particles with 20 h treatment were mixed with the phenolic resin by mechanical stirring for 2 h according to the mass ratio as shown in Table 1. Finally, the mixture was put into a mold for compression molding to obtain the surface-decorated ZrB_2_–SiC-modified phenolic composite. For comparative analysis, an undecorated ZrB_2_–SiC-modified phenolic composite was also fabricated. The initial size of the sample prepared by compression molding was 100 mm × 100 mm × 10 mm.

### 2.3. Preparation of Surface-Decorated ZrB_2_–SiC-Modified C–Ph Composites

The surface-decorated ZrB_2_–SiC particles were mixed with the phenolic resin by mechanical stirring for 2 h, according to the mass ratio as listed in Table 2. The ZrB_2_–SiC particles used for the 1B-C–Ph sample were the untreated ZrB_2_–SiC particles. The surface-decorated ZrB_2_–SiC particles used for the 1C-C–Ph and 2C-C–Ph samples were the particles after 20 h treatment with APS. Subsequently, the carbon fabric was impregnated with the mixture. Then, the prepregs were dried for 6 h in an oven at 80 °C. Finally, the dried prepregs were cut into pieces of 100 mm × 100 mm and were stacked up to fabricate in a vulcanizing machine (Huabo Machinery S&T (Qingdao) Co., Ltd., Qingdao, China) by compression molding. For comparative analysis, the unmodified C–Ph composite was also fabricated. The initial size of the sample prepared by compression molding was 100 mm × 100 mm × 10 mm.

### 2.4. Characterization

The cross-sectional morphologies of the composites before and after ablation were observed by scanning electron microscopy (SEM, HITACHI S4800, Tokyo, Japan), equipped with an energy-dispersive spectroscope (EDS, HITACHI, Tokyo, Japan). The surface three-dimensional shape of the composite after ablation was obtained by 3D microscope (VHX-2000, KEYENCE, Osaka, Japan). The phase characteristics of the residue were determined by X-ray diffraction (XRD, Karlsruhe, Germany). The molecular structure of the surface-decorated ZrB_2_–SiC was detected by Fourier transform infrared spectroscopy (FTIR, Nicolet, Nexus670, Palo Alto, CA, USA). The mechanical property of the composites was evaluated on a universal testing machine (WD-1, Changchun, China) according to GB/T6569-2006. The size of the sample was 3 mm × 4 mm × 36 mm. The strength could be obtained from the following equation:

(1) $$\sigma_{f}=\frac{3FL}{2bd^{2}}$$

where *F* is the maximum compression load at fracture (N), *b* and *d* are the width and thickness (mm) of sample, and *L* is the span of the clamps. Ablation tests were carried out using an oxyacetylene torch. The test sample of Φ30 × 10 mm was subjected to the torch for 30 s. The gas pressures of oxygen and acetylene were 0.7 MPa and 0.05 MPa, respectively. The distance from gun to the surface of sample was 25 mm. The mass ablation ratio was calculated by the following formulas:

(2) $$\mathrm{Mar}=\frac{\Delta m}{\Delta t}.$$

## 3. Results and Discussion

### 3.1. Surface Decoration of ZrB_2_–SiC Particles

**Figure 1:**
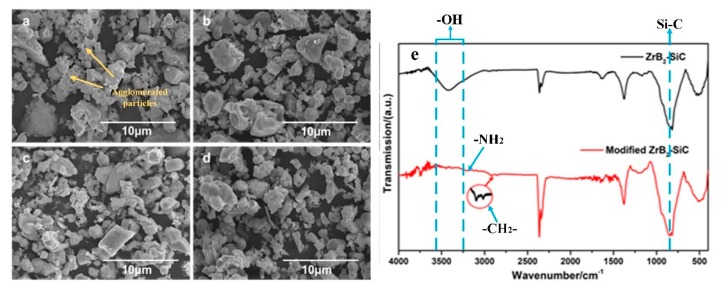
Micro-morphology and FTIR of surface-decorated ZrB_2_–SiC particles treated at different times: (**a**) Untreated, (**b**) 10 h, (**c**) 15 h, (**d**) 20 h, and (**e**) FTIR spectrum of the untreated ZrB_2_–SiC particles and the surface-decorated ZrB_2_–SiC particles with 20 h treatment.

Achieving good surface decoration is the basis for particles possessing good compatibility with the phenolic resin. Figure 1a–d shows the micro-morphologies of the ZrB_2_–SiC particles treated with APS after a different treatment time. As shown in Figure 1a, it can be observed that the untreated ZrB_2_–SiC particles exhibited an obvious agglomeration state with a poor dispersion. The small size particles were adsorbed on the surface of the large particles. After surface decoration, the state of the ZrB_2_–SiC particles changed. When the treatment time was up to 20 h, the agglomeration state of the ZrB_2_–SiC particles disappeared. This is because the organic branches on the surface of the ZrB_2_–SiC particles effectively reduced the surface energy and polarity of the powders, which leads to the improvement of the dispersibility of the inorganic particles.

To further analyze the grafting mechanism of surface-decorated ZrB_2_–SiC particles, the molecular structure of the untreated particles and surface-decorated particles after 20 h treatment were detected by FTIR. As is shown in Figure 1e, all the ZrB_2_–SiC particles before and after surface decoration had absorption peaks at 834 cm^−1^, which are corresponding to the Si–C bond [18]. Meanwhile, the unmodified particles presented a wide hydroxyl absorption peak. However, after treated with APS for 20 h, the hydroxyl absorption peak disappeared. It indicates that the active groups on the surface of the particles have changed significantly. Additionally, two peaks emerged in the range of 2975–2845 cm^−1^, corresponding to the vibrating doublet of methylene [19]. Moreover, an absorption peak existed at 3198 cm^−1^, which was corresponding to the amine group [20]. All these peaks demonstrated the presence of APS organic branches. Therefore, it indicates that the APS has grafted with the hydroxyl groups on the surface of the ZrB_2_–SiC particles. The existence of the organic branches on the surface of the particles provides a prerequisite for achieving good compatibility between the ceramic particles and phenolic resin.

### 3.2. Mechanical Property of the Surface-Decorated ZrB_2_–SiC-Modified Phenolic Composite

The mechanical performance of the ZrB_2_–SiC-modified phenolic composite reflects the modification effect of the surface-decorated particles on the phenolic resin and directly impacts the stability of the material during ablation process. Therefore, investigating the strength of the modified composite is of great significance. The strength of the composite was characterized by the three-point bending test method; the results are listed in Table 3. It is clear that the untreated ZrB_2_–SiC-modified phenolic resin had the lowest strength, which was 69.69 MPa. After being modified with the surface-decorated ZrB_2_–SiC particles, which were treated with APS by 20 h, the strength of the composite was effectively improved. As the content of the particles increased, the bending strength of the material increased constantly. When the mass ratio was up to 1:3, the composite had the highest strength, up to 89.91 MPa. It indicated that the surface decoration of the particles has a positive influence on the mechanical property of the composite.

**Figure 2:**
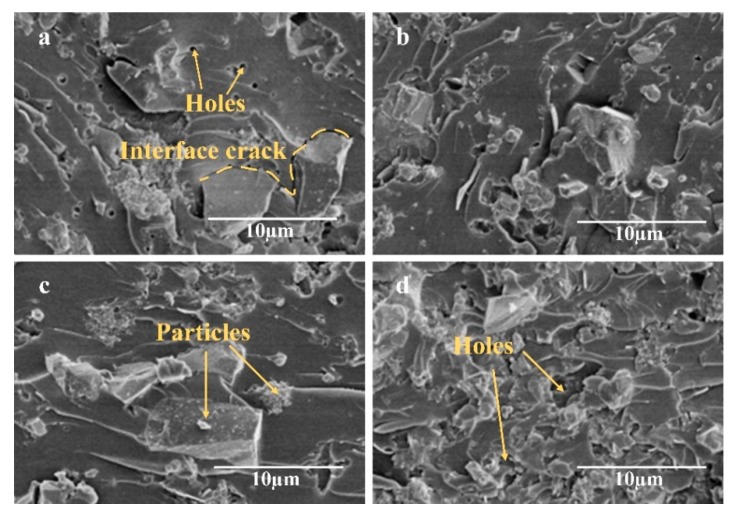
Micro-morphology of ZrB_2_–SiC-modified phenolic composite: (**a**) Unmodified ZrB2–SiC-modified phenolic composite, (**b**) C1 sample, (**c**) C2 sample, and (**d**) C3 sample.

When the content of surface-decorated ZrB_2_–SiC continuously increased to a mass ratio of 1:2, the bending strength was reduced. To analyze the reason why the mechanical performance of the composite has this change. The micro-morphology of the phenolic composite modified by ZrB_2_–SiC particles before and after surface decoration is shown in Figure 2. As shown in Figure 2a, it was obvious that the untreated ZrB_2_–SiC particles had poor compatibility with the phenolic resin. There were some distinct interface cracks between the particles and the resin. Moreover, some holes existed in the resin. During the bending test process, these defect areas became the stress concentration area, which directly led to the fracture failure of the material. The surface-decorated ZrB_2_–SiC-modified phenolic composite was shown in Figure 2b,c, the particles dispersed in the phenolic resin uniformly and the composite formed a dense structure. There were no obvious defects, such as cracks or holes in the interiors of composites. Furthermore, the existence of flexible groups, which comes from the silane coupling agent, could facilitate the absorption of the impact energy and improve the bonding strength of the composite. Meanwhile, after surface decoration by APS, the resin and the surface-decorated particles formed a chemical bond due to the newly created functional groups [21]. All these lead to the improvement of bending strength. However, when the mass ratio reached 1:2, the formability of the composite decreased obviously. Some distinct internal defects appeared, and the composite possessed a non-uniform structure with a large number of holes as illustrated in Figure 2d. This is mainly because that the presence of large amounts of inorganic particles blocking the cross-linking movement of the phenolic molecular during the curing process, which affected the formation of resin. Moreover, the increased viscosity of the mixture decreased the dispersion degree of the particles, which led to the formation of internal defects. Therefore, the bending strength was reduced.

### 3.3. Ablation Behavior of the Surface-Decorated ZrB_2_–SiC-Modified C–Ph Composite

Oxyacetylene torch ablation test was carried out to test the ablation behavior of the C–Ph composite. The mass ablation ratio of four composites is listed in Table 4. We can observe that the unmodified C–Ph had the highest mass ablation ratio, up to 4.61 × 10^−2^ g·s^−1^. It suggests that the unmodified C–Ph composite had a high ablation degree. However, after modification with ZrB_2_–SiC, the mass ablation ratio was reduced. Moreover, with the same content of surface-decorated ZrB_2_–SiC, the mass ablation ratio of the 1C-C–Ph composite was similar to that of the 1B-C–Ph composite. It suggests that the surface decoration of the particle has little influence on the ablation resistance of the composite. When the content of the surface-decorated ZrB_2_–SiC was 25 wt %, the ratio was reduced by 25% compared with that of the unmodified C–Ph composite. This demonstrates that the addition of ZrB_2_–SiC particles itself effectively improved the oxidation ablation performance of the composite.

**Figure 3:**
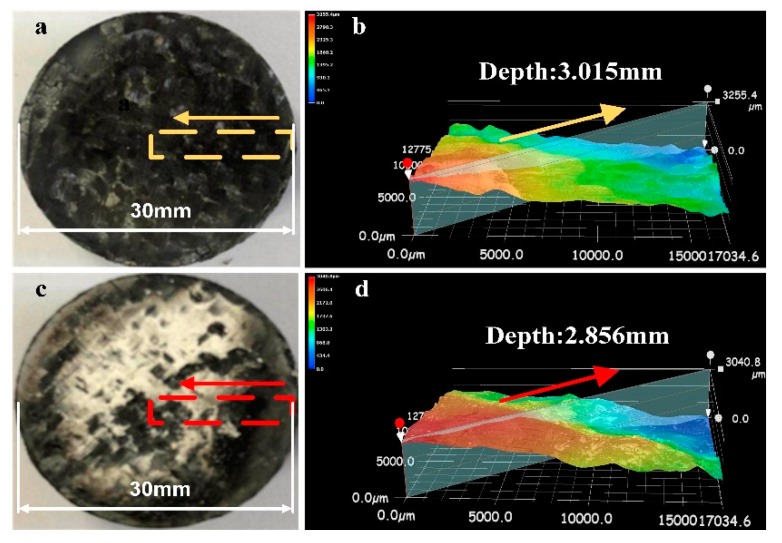
Ablation morphology of the composite: (**a**) Unmodified composite, (**b**) 3D image of the unmodified composite, (**c**) the 2C-C–Ph composite, and (**d**) 3D image of the 2C-C–Ph composite.

To further analyze the ablation behavior of the composite, the unmodified composite and 2C-C–Ph composite were chosen to characterize the macro ablation response. The ablated morphology of these two composites is shown in Figure 3. The unmodified sample exhibited an obvious ablation morphology as illustrated in Figure 3a. The carbon fabric was exposed, and a distinct ablation pits appeared on the central surface of the composite. Moreover, due to the serious oxidative and mechanical erosion resulted from the combustion, the depth of the ablation pit was up to 3.015 mm. However, with the modification of the surfaced-decorated ZrB_2_–SiC particles, the ablation morphology of the composite changed. There was a large amount of white accumulations that covered the ablated surface, as shown in Figure 3c. With the protection of these white accumulations, the depth of the ablation pit was reduced. All these indicate that the presence of the surfaced-decorated ZrB_2_–SiC particles changes the ablation behavior of the composite.

**Figure 4:**
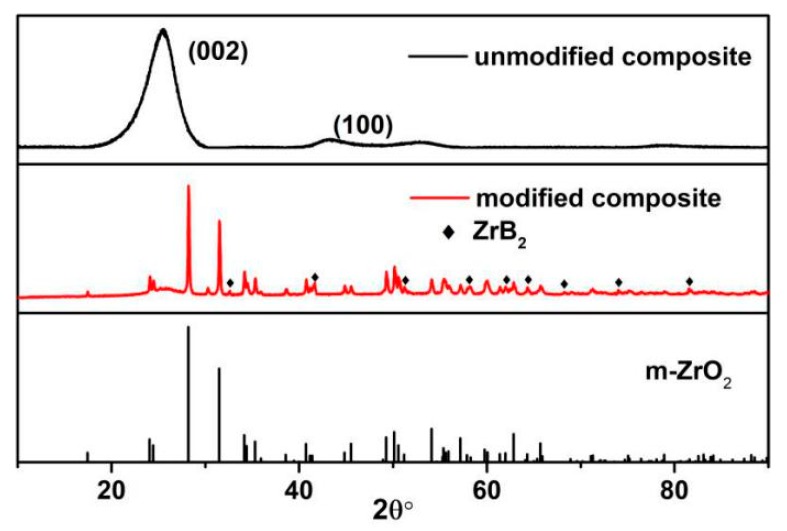
XRD pattern of the composite after ablation.

XRD was used to analyze the phase compositions of the residue on the surface of the composite. The X-ray diffraction patterns are shown in Figure 4. The top curve is the pattern of the unmodified composite. Two broad peaks appeared at nearly 23° and 44°, corresponding to the (002) and (100) lattice planes of graphite, respectively [22,23]. It indicates that the residual char underwent a certain degree of graphitization transformation at high temperature [24]. As for the middle curve related to the 2C-C–Ph composite, the pattern incurred an obvious change, whereby the broad peaks disappeared and were replaced by the characteristic peaks of m-ZrO_2_. Moreover, there was also some characteristic peaks of ZrB_2_. All these suggest that the main phase composition of the white accumulations is m-ZrO_2_ while some unoxidized ZrB_2_ remained. We can conclude that ZrB_2_ is continuously oxidized to supply the zirconia during the ablation, which effectively avoids the loss of ZrO_2_ caused by mechanical erosion. However, as we all know, the oxidation products of ZrB_2_–SiC were ZrO_2_, SiO_2_ and B_2_O_3_. Within the ultrahigh ablation temperature, the B_2_O_3_ incurred serious volatilization. Meanwhile, SiO_2_ was melted and volatilized and some remains existed in the form of a glass phase. Therefore, it cannot be detected by the XRD.

### 3.4. Ablation Mechanism of the Surface-Decorated ZrB_2_–SiC-Modified C–Ph Composite

**Figure 5:**
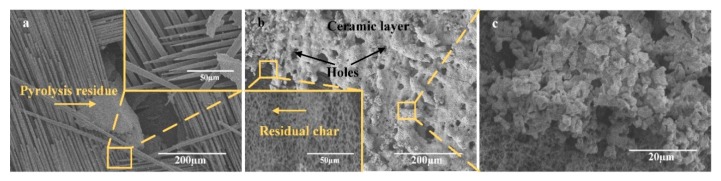
Micro-morphology of composites after ablation: (**a**) Unmodified composite, (**b**) 2C-C–Ph composite, and (**c**) magnification image of the ceramic layer of the 2C-C–Ph composite.

The micro-morphology of the composites after ablation is shown in Figure 5. Figure 5a shows the morphology of unmodified composite, and we can observe that the reinforced carbon fabric was retained on the surface of the composite with a staggered arrangement. Due to the mechanical and oxidative erosion of the flame flow, the surface residual chars almost disappeared. Moreover, the carbon fiber bundles were separated, which led to the inner matrix resin being directly exposed to the flame flow. Therefore, some residual chars pyrolyzed from the inner resin was interposed between the carbon fibers. From the magnification image as shown in Figure 5a, the carbon fiber showed an obvious oxidative morphology with a needle-like structure. It indicates that, during the ablation process, the composites underwent a severe oxidative ablation and mechanical erosion caused by the flame.

After modified with the surface-decorated ZrB_2_–SiC particles, the micro-morphology was changed. As shown in Figure 5b,c, an obvious ceramic layer covered the surface of the ablated region. Under the ceramic layer, the residual char existed with a three-dimensional network structure. Although, parts of the surface ceramic accumulations were peeled off; the retained ceramic layer effectively decreased the mechanical and oxidative erosion on the inner material. Moreover, the porous structure of the residual char formed by the evaporation of the pyrolysis gas, such as H_2_O, CO, CO_2_ and CH_4_, acted as a poor thermal conductor, which effectively obstructed the heat transmission. From the magnification image of the ceramic layer, we can observe that the accumulations possessed an obvious sintered morphology. Combined with the XRD results, we can conclude that the m-ZrO_2_ has incurred sintering to form a dense ceramic layer. However, some holes also existed in the ceramic layer, which resulted from the volatilization of B_2_O_3_ and silica. Moreover, the volatilization of the low melting point oxides can effectively enhance the heat dissipation during the ablation process. Therefore, all these indicate that the addition of the ZrB_2_–SiC particles can improve the ablation resistance of the composite.

**Figure 6:**
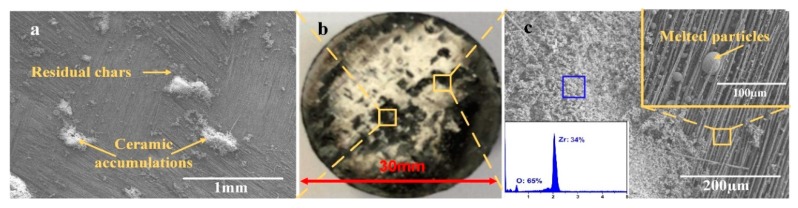
The ablated morphology of the 2C-C–Ph composite at different region: (**a**) The area without ceramic accumulations, (**b**) the 2C-C–Ph composite, and (**c**) the edge area of ceramic accumulations.

From the above analysis, we can conclude that the ablation behavior of the modified composite has changed significantly. To deeply analyze the anti-ablation mechanism of the modified composite, a detailed analysis of the composite ablated surface was made. Figure 6 shows the micro-morphology of the area without ceramic accumulations and the edge area of the ceramic accumulations. As shown in Figure 6a, in the area where the surface ceramic layer was eroded by the flame flow, the carbon fabrics was exposed with a little ceramic accumulations and residual chars. What was different with the unmodified composite, these carbon fibers still possessed good arrangement and the bundles were tight. It suggests that the ceramic layer can effectively decrease the surface erosion degree of the composite. At the edge area, we can observe that the thickness of the accumulations gradually decreased. Through the EDS analysis, the ceramic accumulations were composed with O and Zr. It suggests that there is no silicon presented on the surface of the ceramic accumulations. Combined with the XRD analysis, it can be proved that the accumulations were m-ZrO_2_. In the bare fiber area, there were many melted particles that were interposed between the carbon fibers. Moreover, from the magnification image as shown in Figure 6c, no obvious oxidative morphology existed on the surface of the carbon fabrics. Some melted ceramic accumulations covered on the surface of the fibers, which effectively provided an effective protection for the carbon fibers. Therefore, we can conclude that the formed ceramic layer plays an essential role on the anti-oxidative ablation performance of the composite.

**Figure 7:**
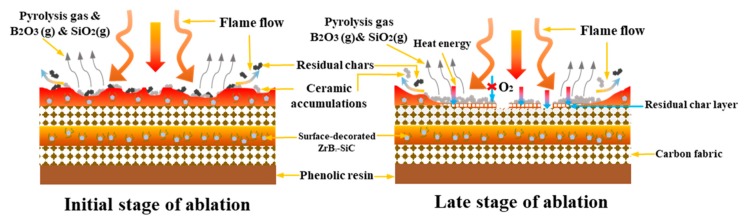
Diagram of the underlying ablation resistance mechanism of surface-decorated ZrB_2_–SiC-modified composites.

To further reveal the anti-ablation mechanism of the surface-decorated ZrB_2_–SiC-modified composite, a diagram model was established on the basis of the above analysis. As shown in Figure 7, in the initial stage of ablation, the flame flow directly led to the pyrolysis of the matrix resin and oxidation of the inorganic particles. With the deposition of heat energy, the oxidative products, such as B_2_O_3_ and SiO_2_, were melted and volatilized. Combined with the pyrolysis gas formed by the matrix resin, it can effectively improve the heat dissipation. Moreover, due to the serious mechanical and oxidative erosion of the flame flow, the residual chars and ceramic accumulations were eroded. As the ablation time increase, the ablation degree was enhanced. The ceramic accumulations were continuously generated and sintered, which covered the surface of the composite. With the volatilization of the B_2_O_3_ and SiO_2_, the main composition of the ceramics was m-ZrO_2_. Because the oxygen diffusion index of m-ZrO_2_ is 10^−9^ g/cm·s [25], it can act as a good oxygen barrier to prevent the oxidative flame from the inner material. Meanwhile, with the protection of the ceramic layer, the inner residual chars were retained, which can act as a thermal insulator and effectively obstruct the heat transmission [26]. However, some ceramic accumulations and residual chars were eroded by the mechanical erosion of the flow, and the inner carbon fabrics was exposed to the flame. Meanwhile, with the coverage of the ceramics, the carbon fabrics still kept their arrangement without oxidative ablation. Therefore, the ZrB_2_–SiC-modified C–Ph composite has a good anti-ablation performance.

## 4. Conclusions

Surface-decorated ZrB_2_–SiC-modified phenolic composites and carbon fabric reinforced phenolic composites were fabricated by thermal compression molding. The mechanical property and ablation resistance of the composites were characterized. It was observed that the organic branches were effectively retained on the surface of the particles with the treatment of APS for 20 h. The agglomeration degree of the particles was reduced and the dispersibility of particles in the phenolic resin was improved. The organic branches of the surface-decorated ZrB_2_–SiC enhanced the interface performance between the resin and particles. The mechanical property of the surface-decorated ZrB_2_–SiC-modified phenolic composites was enhanced by 13%, compared with the same content ZrB_2_–SiC-modified phenolic composite. Moreover, the surface decoration has no significant effect on the ablation resistance of composites. The existence of the ZrB_2_–SiC particles improved the ablation resistance of the C–Ph composite. The mass ablation ratio was reduced by 25%, compared with the unmodified C–Ph composite. The formed ZrO_2_ layer covered the surface of the composite, effectively preventing the heat and oxygen from penetrating into the interior. Meanwhile, the volatilization of the molten SiO_2_ and B_2_O_3_ combined with the pyrolysis gas effectively dissipated the heat energy and further improved the ablation resistance of the composite. Thus, the surface-decorated ZrB_2_–SiC can effectively improve the interface properties between particles and phenolic resin and the particles itself can effectively enhance the anti-oxidative ablation performance of the composite.

## Figures and Tables

**Table 1:** The mass proportion of the samples.

Sample	APS/mL	H_2_O/mL	Ethanol/mL	ZrB_2_–SiC/g	ZrB_2_–SiC/Phenolic Mass Ratio
B1	0	0	0	20	1:4
C1	12	8	80	20	1:4
C2	12	8	80	20	1:3
C3	12	8	80	20	1:2

**Table 2:** The mass proportion of the C–Ph composite samples.

Sample	ZrB_2_–SiC/Phenolic Mass Ratio	Phenolic/Carbon Fabric Volume Ratio
Unmodified Composite	0	1:1
1B-C–Ph	1:4	1:1
1C-C–Ph	1:4	1:1
2C-C–Ph	1:3	1:1

**Table 3:** Bending strength of the ZrB_2_–SiC-modified phenolic composites.

Sample	ZrB_2_-SiC/Phenolic Volume Ratio	Bending Strength/MPa
B1	1:4	$69.69_{-2.5}^{+1.7}$
C1	1:4	$77.66_{-1.8}^{+2.4}$
C2	1:3	$89.91_{-1.9}^{+2.2}$
C3	1:2	$70.36_{-2.4}^{+3.2}$

**Table 4:** Mass ablation ratio of the modified C–Ph composites.

Sample	Mass Ablation Ratio/(10^−2^ g·s^−1^)
Unmodified Composite	4.61
1B-C–Ph	3.88
1C-C–Ph	3.87
2C-C–Ph	3.46
